# Perceived safety climate in Irish primary care settings—a comparison with Scotland and England

**DOI:** 10.1080/13814788.2018.1524002

**Published:** 2018-11-01

**Authors:** Ciara Curran, Sinéad Lydon, Maureen E. Kelly, Andrew W. Murphy, Caoimhe Madden, Paul O’Connor

**Affiliations:** a Discipline of General Practice, School of Medicine, National University of Ireland Galway, Galway, Ireland;; b Irish Centre for Applied Patient Safety and Simulation, National University of Ireland Galway, Galway, Ireland;; c HRB Primary Care Clinical Trials Network Ireland, National University of Ireland—Galway, School of Medicine, Galway, Galway, Ireland

**Keywords:** Patient safety, safety climate, workload, primary care, general practice

## Abstract

**Background:** Safety climate (SC) measurement is a key component of quality and safety initiatives in primary healthcare.

**Objectives:** To (1) measure perceived SC in Irish primary care; (2) examine whether perceptions of safety varied according to respondent characteristics; and (3) compare responses from our Irish sample to data from England and Scotland.

**Methods:** PC-SafeQuest Survey was administered to all practice staff in Irish general practices between March and May 2016. This survey consists of 30 items across five safety domains (workload, communication, leadership, teamwork, and safety systems). Multiple regression analysis was used to identify predictor variables of perceived safety. The effect size of the difference between the Irish sample’s scores and published English and Scottish data were calculated.

**Results:** A total of 231 questionnaires (38.5%) were returned. Generally, positive perceptions of perceived safety were identified among Irish respondents, but workload had the lowest overall mean score (M = 4.3, SD = 1.2) of the five domains. Comparisons across the Irish, English and Scottish samples identified a medium size effect difference in workload; Scottish respondents perceived workload to have less of a negative impact on safety than Irish or English counterparts (Cohen’s *d* = 0.602, 0.67 respectively). Analyses indicated that Irish GP principals perceived a more negative impact of workload on safety than administrative staff (*β* = 0.28, *P* = 0.03).

**Conclusion:** Irish SC data are largely similar to those of England and Scotland. The perceived potential for workload to negatively impact upon safety emerged within each country. In Ireland, GP principals perceive this as a greater threat than practice administrators do.

Key messages

- Safety climate measurement has become a key component of patient safety toolkits in primary care.
- When safety climate data from Ireland were compared to data from England and Scotland, the results were broadly similar—perceptions of safety were generally positive but primary care staff perceived a potential for workload to impact negatively upon patient safety.

## Introduction

Safety climate (SC) has been recommended as a useful component of quality and safety improvement initiatives in healthcare [1,2]. SC is regarded as the measurable component of safety culture [3], which is defined as the values, attitudes, norms, beliefs, practices, policies, and behaviours about safety issues in daily practice [4]. Positive perceptions of SC are associated with an open culture and willingness to learn from mistakes and errors [5]. Hospital-based research has indicated that perceived SC is positively associated with clinical outcomes and the safety behaviours and attitudes of staff [6].

In secondary care, SC data have been used to benchmark safety cultures internationally [7]. This process has resulted in the identification of systematic differences in perceived SC within and between hospitals, allowing for the identification of specific areas of safety that may be targeted for improvement [7]. A recent systematic review [8] found that in primary care, SC instruments are rarely used beyond the initial development study, suggesting that there is a need to investigate SC across primary care organizations internationally.

The comparison of SC perceptions across employees within organizations can also yield useful data. In primary care settings, differences in SC perception by gender and job position (clinical versus non-clinical) have been inconsistent across studies [9–13]. However, it has been repeatedly found that respondents in management roles (e.g. general practitioner (GP), principals/practice managers) report significantly more positive SC perceptions than those performing non-management roles (e.g. GP assistants, nurses, administrators) [9,10,13]. The explanation for this finding is that managers may be detached from front-line operations, resulting in more favourable perceptions of staff and system safety performance [9].

The purpose of this study is to assess perceived SC in Irish primary care and to examine whether perceptions of safety-related domains varied significantly according to respondent characteristics. Furthermore, perceived SC within Irish primary care practices will be compared to SC data from English and Scottish samples [9,13]. An international comparison of perceived SC across Ireland, Scotland and England is possible, as both the studies from Scotland and England are the only known published studies to utilize the same SC survey instrument [14] that will be used to measure SC in this study [9,13].

## Methods

### Setting and sample size

Staff in 187 practices in the Western Research and Education Network (WestREN) were surveyed as part of the study. WestREN is a university-affiliated general practice research network in the west of Ireland that has been shown to be broadly representative of the national general practice profile in Ireland [15].

#### Data collection

Ethical approval for this study was obtained from the Irish College of General Practitioners (1 May 2015). Following approval, general practices affiliated with the WestREN were invited by letter or email to participate in the ‘NUI Galway WestREN Safety Climate Survey’ from April 2016. GP principals or practice managers distributed the surveys to clinical and administrative staff. Practice staff completed the surveys anonymously. Surveys were returned either in a prepaid envelope or electronically to the research team. Reminders were sent two and four weeks after initial contact.

### Survey instrument

Perceived SC was measured using the PC-SafeQuest (Primary Care-SafeQuest). This instrument was developed specifically for SC assessment in primary care and has good reliability and validity [8,14]. PC-SafeQuest has 30 items, which measure five specific SC domains:

- ‘Workload’—perception of the effects of working conditions on patient safety.
- ‘Communication’—perceived importance of open and honest discussion between practice team members.
- ‘Leadership’—perception of management’s commitment to safety within the practice.
- ‘Teamwork’—perception of the importance of collaboration between practice team members to deliver efficient and effective patient care.
- ‘Safety systems’—perception of engagement with risk assessment and safety management techniques by practice team members.

Responses are measured on a seven-point scale from 1 (‘not at all’) to 3 (‘to a limited extent’) through to 7 (‘to a very great extent’). Higher scores on each of the domains are indicative of a perception that the factor contributes positively to safety in the practice while lower scores on a domain are indicative of a perception that the factor is perceived to detract from safety within the practice to an extent.

A demographic questionnaire was used to collect information on the following respondent characteristics: gender, job role, work pattern, the number of years of experience in the practice and total number of years’ experience in general practice.

### Statistical analysis

All data were entered into SPSS (version 22; IBM Corporation, Armonk, NY, USA) for analysis. Negatively worded items were reverse scored for analysis purposes. Preliminary analyses were conducted to ensure the assumptions of normality, linearity, multicollinearity, and homoscedasticity were within acceptable limits. Missing data (1.92%) were managed by excluding cases pairwise. Following this initial screening, a series of six multiple linear regressions were conducted to examine whether demographic variables were predictive of ‘favourable’ SC domain scores and/or the overall SC score. These multiple regressions, using a forced entry method, were carried out with the following predictor variables: gender (male, female); role within the practice (GP principal, GP other, nurse, administration); years of professional experience (<10 years, > 10 years); years of experience within this practice (<10 years, > 10 years); work pattern (full-time, part-time); and practice staff characteristics (management, non-management). Both ‘role within the practice’ and ‘practice staff characteristics’ were included within the regressions to assess whether differences in perceptions of safety existed according to professional role, and to facilitate the comparison of our data with international data suggesting differences in perceptions of safety between managerial and non-managerial staff [9,10,12,13].

Cohen’s *d* was used to measure the effect size of the standardized mean difference between the Irish sample [16], a sample of 563 primary care respondents from Scotland [9], and 335 respondents from England [13]^.^ Effect sizes are generally classified as small (Cohen’s *d* = 0.2), medium (Cohen’s *d* = 0.5) and large (Cohen’s *d* = 0.8), where greater than a medium effect size is generally of practical significance [16]. Both the Scottish and English studies utilized the PC-SafeQuest questionnaire and were reported in the literature in 2012 and 2015, respectively [9,13].

## Results

### Response rate and demographics

Participating practices of the WestREN network requested 600 surveys of which 231 were returned (38.5% response rate). Most respondents were female (71.2%), and worked full-time (63.5%). Other respondent characteristics and reported demographics of participants from the English and Scottish data samples are provided in Table 1 [9,13].

**Table 1.** Respondent characteristics across all three data samples.

Characteristics	Ireland	Scotland [9]	England [13]
	*n* (%)	*n* (%)^a^	*n* (%)
Professional role			
Managerial	98 (45.2)	208 (36.9)	102 (30.4)
Non-managerial	119 (54.8)	343 (60.9)	233 (69.6)
Gender			
Male	62 (28.6)	92 (16.4)	56 (16.7)
Female	155 (71.4)	467 (83.5)	279 (83.3)
Work pattern			
Full-time	138 (63.5)	280 (49.7)	195 (41.4)
Part-time	77 (35.6)	281 (49.9)	138 (58.6)
Locum	2 (0.9)		
Years in current practice			
<10 years	109 (50)	331 (59.2)	213 (63.6)
>10 years	111 (50)	228 (40.8)	122 (36.4)
Years of experience in primary care			
<10 years	88 (40.6)		171 (51)
>10 years	129 (59.4)		164 (49)

a Missing data has been accounted for in this column [9].

### Reliability of the survey instrument

The Cronbach’s alpha coefficient of scale reliability was 0.82 for the overall SC score, which is considered to be acceptable. Cronbach’s alpha scores for each of the subscales were also favourable (for workload = 0.69; communication = 0.89; leadership = 0.71; teamwork = 0.93; safety systems = 0.90) [17].

### Perceptions of safety climate in Ireland

Table 2 shows the mean and standard deviations for each survey domain. The ‘leadership’ domain had the highest mean score, and ‘workload’ had the lowest. Closer examination of responses to individual items in the workload domain showed that two items with particularly negative responses were ‘team members always have enough time to complete work tasks safely’ (modal response is 3 ‘agree to a limited extent’), and ‘when pressure builds up, team members are expected to work faster even if it means taking shortcuts’ (modal response is 7 ‘to a very great extent’).

**Table 2.** Safety climate scores from Ireland, Scotland and England, and comparison of scores across the countries using effect sizes.

	Ireland		Scotland		England		Ireland-Scotland	Ireland-England	Scotland-England comparison
							Comparison	Comparison	
Domain	Mean^a^	SD	Mean^a^	SD	Mean^a^	SD	Cohen’s *d*^b^	Cohen’s *d*^b^	Cohen’s *d*^b^
Workload	4.3	1.2	5.0	1.2	4.2	1.2	−0.6	0.1	0.7
Communication	5.4	1.3	5.1	1.1	4.7	1.4	0.3	0.5	0.3
Leadership	6.1	0.9	6.1	0.9	5.5	1.3	0	0.5	0.5
Teamwork	6.0	1.0	5.7	0.9	5.3	1.2	0.3	0.6	0.4
Safety systems	5.5	1.1	5.6	1.07	5.5	1.2	−0.1	0	0.1
Overall	5.4	0.8	5.5	0.8	5.1	1.0	−0.1	0.3	0.4

a Higher scores with each of the domains are indicative of a perception that the factor contributes positively to safety in the practice while lower scores on a domain are indicative of a perception that the factor detracts from safety within the practice

b Cohen’s *d* represents an effect size. An effect size quantifies the size of the difference between two groups and it may be considered to be a true measure of the significance of the difference. Cohen’s *d* effect sizes are generally classified as small (*d* = 0.2), medium (*d* = 0.5), and large (*d* = 0.8), where greater than a medium effect size is generally of practical significance [16].

### Perceptions of safety climate according to respondent characteristics

Only the workload domain resulted in a significant regression model (*F* (8, 206) = 2.38, *p* = 0.02, *R*^2^ = 0.085). Workload scores were significantly higher among administrative staff (receptionist/practice managers) as compared to GP principals (*β* = 0.28, *p* = 0.03). As higher scores are indicative of a more positive disposition to the domain, this finding suggests that GP principals perceived a significantly more negative impact of workload on safety and performance within the practice than administrative staff.

### Comparisons of safety climate between Ireland, Scotland and England

There was little difference between the overall SC scores for comparisons of Irish, Scottish and English samples (see Table 2). However, Scottish respondents perceived less of a negative impact of workload on safety than both Irish (Cohen’s *d* = −0.6) and English (Cohen’s *d* = 0.7) respondents (see Table 2). For the leadership domain, the Irish and Scottish perception of the impact of leadership on work performance and safety was more positive than the English sample (both Cohen’s *d* = 0.5; see Table 2). A medium-sized difference (Cohen’s *d* = 0.6) was also reported between Irish and English samples across the teamwork domain, indicating that teamwork was perceived as less likely to compromise practice and patient safety by Irish respondents.

## Discussion

### Main findings

Overall, SC was perceived to be largely positive within Irish primary care and was found to be broadly comparable to England and Scotland. Across all three samples, workload was perceived to have the potential to negatively affect patient safety and received the lowest mean SC domain scores. In the Irish sample, GP Principals perceived a significantly more negative impact of workload on safety and performance within the practice than perceived by administrative staff.

### Strengths and limitations

The internal reliability was found to be acceptable for the overall questionnaire score and domain scores. Responses were obtained from GPs and other practice staff with a broad range of experiences allowing for comparisons in attitudes to SC to be compared based upon a range of respondent characteristics.

However, recruiting respondents through the university-affiliated network could also be considered a limitation as it may have introduced a voluntary response bias. Practices within the university network may theoretically be more likely to be responsive to the survey invitation or practices that did respond to the survey may be more interested in patient safety than non-responders. As we anonymized the results to encourage responses confidentially, we had no data available to allow comparison of responders to non-responders.

Next, the response rate (38.5%) may affect the external validity of our results. However, this was higher than the 29% respondent rate of the similar English study [13].

Finally, the delay in publication of the data, collected in 2016, may also be considered a limitation. However, there has been no notable shift in the context or nature of primary healthcare in Ireland, Scotland or England, and there is no reason to indicate that perceived SC would have changed during this period.

### Comparison with existing literature

*Workload*. There has been a substantial, and unaddressed, increase in workload in primary care [18]. All three data samples (Ireland, England and Scotland [9,13]) within this paper reported the lowest mean domain score for workload, suggesting a perception that it was likely to impact on safety negatively. A perceived negative impact of workload on SC and patient safety has been reported in other European countries and the US [10,19]. A recent survey of GPs in the UK reported that more than nine out of ten GPs believe that their workload has negatively affected quality of care [20]. Further, research has suggested that excessive workload in primary care is linked to almost half of adverse events and near misses [21]. Across our three samples, Scottish respondents perceived workload as less of a threat to patient safety than Irish or English respondents did [9]. It has been found previously that, within the UK, GPs in Scotland were least likely to say their workload was both ‘unmanageable’ and that it had ‘significantly negatively impacted on the quality of care that patients had received’ [20]. Further reflection on Scottish and other European primary care practices, where GPs have a better perception of the impact of workload on their daily practice, may be worthwhile [22] if we are to develop interventions to tackle the workload issue internationally and improve safety.

*Differences in safety climate perception based on professional roles.* In contrast to previous European studies [9,10,12,13], we did not find any significant difference between the perception of SC by management/non-management staff roles. The more positive managerial SC perceptions reported previously had been attributed to detachment from frontline operations [9,10,12,13]. One possible reason for our finding may be the smaller team size in Ireland than in the UK—the average number of GPs per practice in the WestREN is 2.4 GPs compared to 6 or 7 in England [15,22], and more than 90% of practices in the UK are considered as group practices [22]. With smaller practice teams, staff are likely to work more closely and cohesively with each other [12] and it is arguable that detachment from the front-line interface is less likely to occur.

*International comparison of safety climate.* Perceptions of SC in primary care in other European countries have also been found to be generally positive [10–12]. However, when benchmarking SC results across primary care settings, it is important to take into account contextual differences in primary healthcare delivery [23]. Irish GPs typically work in a mixed public–private system, as opposed to state-led universal healthcare system such as the National Health Service (NHS) which operates in Scotland and England [22]. There was also a notable positive perception of the impact of leadership and teamwork on work performance and safety noted by Irish respondents. It is arguable that greater independence and autonomy associated with private practice in Ireland [24] may positively influence perceptions of SC. However, new government-led healthcare delivery models in Ireland involve the development of large primary care centres [25], which will likely increase the average size of practices in Ireland. Therefore, it will be important to foster shared cultural perceptions about SC and the delivery of safe high-quality care in these larger practices so that staff members continue in close alignment with one another.

### Implications for future research and clinical practice

Based on our findings, we offer the following recommendations for future research and practice:

- Participation in SC measurement may increase awareness of safety at an individual team member level [9]. At a practice level, SC measurement can be used as an educational tool to identify relative strengths and weaknesses, which may, in turn, be targeted by initiatives to build a stronger safety culture [9]. SC measures have been widely used in primary care in the Scottish Patient Safety Programme in Primary Care [9], where a high usage of the intervention has resulted in an improved quality of, and safer, patient care [26].
- There is a need for more detailed assessment of the contributors to workload in primary care and of how these factors can be addressed and alleviated, to develop interventions to improve the delivery of safe and efficient patient care.
- SC measures have been benchmarked across healthcare systems in different geographical areas, and countries, in secondary care with aggregation of data informing safety and educational opportunities [6,7]. There is a need to consider benchmarking SC measures at a primary care level, as in the current study, to inform and target safety improvements. Patient safety programmes must deal with specific characteristics of primary care and careful consideration should be given to contextual primary healthcare settings when interpreting results of SC surveys [8,23].

## Conclusion

In Irish primary care, perceived SC was generally positive and broadly similar to published data from England and Scotland. All three studies highlighted the negative impact of workload on perceived SC. In Ireland, GP principals perceive this as a greater threat than practice administrators do.

## References

[1] National Patient Safety Agency Seven steps to patient safety. London: NPSA, (Second Print August) 2004-07 (V1).

[2] Royal College of General Practitioners. Patient safety toolkit for general practice (internet); London: RCGP; 2017 Available from: http://www.rcgp.org.uk/clinical-and-research/toolkits/patient-safety.aspx; [Cited 2018 July 15].

[3] Huang Y-h, Zohar D, Robertson M, et al. Development and validation of safety climate scales for lone workers using truck drivers as exemplary. Transportation Res Part F: Traffic Psychol Behav. 2014;26:348–360.

[4] Mearns KJ, Flin R Assessing the state of organizational safety—culture or climate. Curr Psychol. 1999;18:5.

[5] Zohar D, Livne Y, Tenne-Gazit O, et al. Healthcare climate: a framework for measuring and improving patient safety. Crit Care Med. 2007;35:1312–1217. 17414090 10.1097/01.CCM.0000262404.10203.C9

[6] Nieva VF, Sorra J Safety Culture assessment: a tool for improving patient safety in healthcare organisations. BMJ Qual Saf. 2003;12:ii17–ii23. 10.1136/qhc.12.suppl_2.ii17 PMC1765782 14645891

[7] Sorra J, Nieva V, Famolaro T, et al. Hospital survey on patient safety culture: 2007 comparative database report. Rockville (MD): Agency for Healthcare Research and Quality (US); 2007 (Prepared by Westat, Rockville, MD, under contract No. 233-02-0087, Task Order No. 18.) AHRQ Publication No. 07-0025.

[8] Curran C, Lydon S, Kelly ME, et al. A systematic review of primary care safety climate survey instruments: their origins, psychometric properties, quality and usage. J Patient Saf. 2018;14:e9–e18. 28708671 10.1097/PTS.0000000000000393

[9] De Wet C, Johnson P, Mash R, et al. Measuring perceptions of safety climate in primary care: a cross sectional study. J Eval Clin Pract. 2012;18:135–142. 20860593 10.1111/j.1365-2753.2010.01537.x

[10] Astier-Pena MP, Torijano-Casalengua ML, Olivera-Canada G, et al. Are Spanish primary care professionals aware of patient safety. Eur J Public Health. 2015;25:781–787. 25842381 10.1093/eurpub/ckv066

[11] Bondevik GT, Hofoss D, Hansen EH, et al. Patient safety culture in Norwegian primary care: a study in out-of-hours casualty clinics and GP practices. Scand J Prim Health Care. 2014;32:132–138. 25263763 10.3109/02813432.2014.962791 PMC4206561

[12] Hoffmann B, Miessner C, Albay Z, et al. Impact of individual and team features of patient safety climate: a survey in family practices. Ann Fam Med. 2013;11:355–362. 23835822 10.1370/afm.1500 PMC3704496

[13] Bell B, Reeves D, Marsden K, et al. Safety climate in English general practices: workload pressures may compromise safety. J Eval Clin Pract. 2016;22:71–76. 26278127 10.1111/jep.12437 PMC4949509

[14] De Wet C, Spence W, Mash R, et al. The development and psychometric evaluation of a safety climate measure for primary care. Qual Saf Health Care. 2010;19:578–584. 20406910 10.1136/qshc.2008.031062

[15] Kavanagh KE, O’Brien N, Glynn LG, et al. WestREN: a description of an Irish academic general practice research network. BMC Fam Pract. 2010;11:74. 20925958 10.1186/1471-2296-11-74 PMC2958962

[16] Lakens D Calculating and reporting effect sizes to facilitate cumulative science: a practical primer for *t* tests and ANOVAs. Front Psychol. 2013;4:863. 24324449 10.3389/fpsyg.2013.00863 PMC3840331

[17] Bland JM, Altman DG Cronbach’s alpha. BMJ. 1997;314(7080):572. 9055718 10.1136/bmj.314.7080.572 PMC2126061

[18] Hobbs R, Bankhead C, Mukhtar T, et al. Clinical workload in UK primary care: a retrospective analysis of 100 million consultations in England, 2007–14. Lancet. 2016;387:2323–2330. 27059888 10.1016/S0140-6736(16)00620-6 PMC4899422

[19] Famolaro T, Yount D, Hare R. et al. Medical office survey on patient safety culture: 2016 user comparative database report. Rockville (MD): Agency for Healthcare Research and Quality (AHRQ) Available from: https://psnet.ahrq.gov/resources/resource/30119/medical-office-survey-on-patient-safety-culture-2016-user-comparative-database-report. [Cited 2018 July 15]

[20] British Medical Association British Medical Association national survey of GPs ‘the future of general practice 2015’ London: British Medical Association; 2015 [Cited 2018 July 15]. Available from: https://www.bma.org.uk/-/media/files/pdfs/working%20for%20change/negotiating%20for%20the%20profession/general%20practitioners/future%20of%20general%20practice%20full%20survey%202015.pdf

[21] Kostopoulou O, Delaney B Confidential reporting of patient safety in primary care: results from multilevel classification of cognitive and system factors. Qual Saf Health Care. 2007;16:95–100. 17403753 10.1136/qshc.2006.020909 PMC2653163

[22] Mc Carthy M Sustainable general practice: looking across Europe. Br J Gen Pract. 2016;66:36. 26719469 10.3399/bjgp16X683233 PMC4684014

[23] Verstappen W, Gaal S, Esmail A, et al. Patient safety improvement programmes for primary care. Review of a Delphi procedure and pilot studies by the LINNEAUS collaboration on patient safety in primary care. Eur J Gen Pract. 2015;21(sup 1):50–55. 26339837 10.3109/13814788.2015.1043725 PMC4828596

[24] O’ Dea B, O’Connor P, Lydon S, et al. Prevalence of burnout among Irish general practitioners: a cross sectional study. Ir J Med Sci. 2017;186:447–453. 10.1007/s11845-016-1407-9 26803315

[25] Collins C, O’Riordan M The future of Irish general practice: ICGP member survey 2015. Dublin (Ireland): Irish College of General Practitioners; 2015 Available from: https://www.icgp.ie/go/library/catalogue/item/E21F0871-DDC4-1AD4-20024CFC3C37FE68. (Cited 2018 July 15).

[26] Houston N, Bowie P The Scottish patient safety programme in primary care: context, interventions, and early outcomes. Scott Med J. 2015;60:192–195. 26449921 10.1177/0036933015606577

